# Revealing the impact of the Caucasus region on the genetic legacy of Romani people from genome-wide data

**DOI:** 10.1371/journal.pone.0202890

**Published:** 2018-09-10

**Authors:** Zsolt Bánfai, Valerián Ádám, Etelka Pöstyéni, Gergely Büki, Márta Czakó, Attila Miseta, Béla Melegh

**Affiliations:** 1 Department of Medical Genetics, Clinical Centre, University of Pécs, Pécs, Hungary; 2 Szentágothai Research Centre, University of Pécs, Pécs, Hungary; 3 University of Pécs, Medical School, Department of Laboratory Medicine, Pécs, Hungary; Kunming Institute of Zoology, Chinese Academy of Sciences, CHINA

## Abstract

Romani people are a significant minority in Europe counting about 10 million individuals scattered throughout the continent. They are a migratory group originating from Northwestern India. Their exodus from India occurred approximately 1000–1500 years ago. The migration route of the Romani people was reconstructed with the help of cultural anthropology, linguistics and historical records. Their migration made them through Central Asia, Middle East and the Caucasus region, prior to the arriving into Europe. Yet the significance of these regions, especially of the Caucasus, in Roma ancestry was a rather neglected topic. Contribution of the Caucasus and further affected regions to the ancestry of Roma was investigated based on genome-wide autosomal marker data. 158 European Roma samples and 41 populations from the Caucasus region, from Middle East, Central Asia and from South Asia were considered in our tests. Population structure and ancestry analysis algorithms were applied to investigate the relationship of Roma with these populations. Identical by descent DNA segment analyses and admixture linkage disequilibrium based tests were also applied. Our results suggest that the Caucasus region plays also a significant role in the genetic legacy of Romani people besides the main sources, Europe and South Asia, previously investigated by other population genetic studies. The Middle East and Central Asia seems slightly less important but far from negligible in connection with the sources of Roma ancestry. Our results point out that the Caucasus region and altogether the area of the Caspian and Black Seas had a significant role in the migration of Romani people towards Europe and contributed significantly to the genetic legacy of Roma rival to the European and Indian main sources.

## Introduction

The Romani people (Roma, Gypsies) are an itinerant ethnic group with a 10–15 million estimated census size [1] residing mainly in Europe, with the largest numbers concentrated into the East-Central European region [2, 3], but they live in large numbers in the Iberian Peninsula as well [4]. They can also be found in the Caucasus, Middle East and in the Americas. Roma are a diasporic population, without their own written history. The geographically dispersed nomadic Roma populations have been socially excluded and also often persecuted throughout history from the Middle Ages to the present days [5]. There were several attempts also for their forced assimilation, e.g. in the Habsburg Monarchy, Spain or Norway from the 18^th^ to the early 20^th^ century [5, 6].

Studies about the origin of Roma are based on historical, linguistic, anthropological and genetic evidences. Historical records of host countries about Roma have initially suggested that Roma are originating from the Indian subcontinent and they migrated towards Europe in the 5^th^ and 10^th^ centuries [5]. Linguistic and anthropological studies shed light on significant similarities between the language and culture of distinct Indian ethnic groups and that of Roma. The social structure of Roma is very similar to the Indian caste system, where a group, called caste, is often defined by the profession of its members [2, 5]. The endogamic habits, appearing at several Roma subgroups, are also similar to the Indian practices. Although linguistics could not find a connection between Romani people and Banjara from India, a link between the two people have been suggested based on anthropological evidences [5]. However, comparative linguistics have suggested the highest relatedness of Northwestern Indian (Punjabi, Kashmiri) or the Central Indian (Hindi) dialects to the Roma language [7, 8].

Genetic investigations based on the study of paternal and maternal lineages (Y-chromosome markers and mtDNA) confirmed the South Asian origin of Roma [9–12]. However, these studies were contradicting each other, because Y-chromosome studies suggested a South Indian origin, while mtDNA pointed out the Northwest Indian origin of Roma [13]. Studies based on genome-wide autosomal single nucleotide polymorphism (SNP) data determined the source of South Asian and European ancestries of the Romani people, and concluded that Roma are an admixed ethnic group with West Eurasian and South Asian ancestry [14, 15]. These studies determined also the proportions of the two ancestry sources and estimated the date of European gene flow into the ancestors of Roma. The studies placed the origin of Roma to the Northwest region of India, to the states of Punjab, Gujarat, Jammu and Kashmir. A more recent study reinvestigated the topic applying significantly larger sample sizes both in case of Roma and Indian groups. The paper strengthened the Northwest Indian origin of Roma and concluded that Pakistan could also play significant role in the origin of Romani people [16].

The exodus of the Romani people from India began with heading north from the Hindu Kush, and continued by wandering across the Iranian plateau, reaching the southern shores of the Caspian and Black Seas, then heading into Europe through the Bosphorus [4]. Romani wandered through Central Asian, Middle Eastern regions and through the Caucasus area, before reaching Europe in the 12^th^ century. It is known that Roma remained in the Balkans before entering deeper into Europe which was basically driven by the Ottoman conquest campaigns reaching the region. The Roma settled in multiple locations in Europe and were already widespread throughout Europe by the end of the 15^th^ century [4]. Three more recent migrations within Europe played a role in the formation of the dispersal of present-day European Roma populations. The first occurred after the abolition of Roma slavery in the Romanian Old Kingdom in the late 19^th^ century, the second was in the second half of the 20^th^ century from Yugoslavia, and the third was at the beginning of the 2000s when Roma began to migrate from Central and East Europe towards the western parts of Europe. [1, 17, 18]

Regarding their connection to populations on their migration route, linguists have already connected the Middle East and Roma. The English term “Gypsy”, the Spanish “Gitano” or the French term “Gitan” from the Middle Ages reflects a belief that Romani people were migratory Egyptians, which has various interpretations, but ultimately suggests that some groups of the Roma exodus from India migrated to and possibly lived in the Middle East. A genetic study based on the investigation of Y and mtDNA haplogroups suggests a remarkable connection between the Middle East, Central Asia and the Romani people as they discovered a high share of haplogroups in Roma, which can be found in populations of the Middle East and Central Asia. [19].

Here we analyzed Central European Roma populations based on genome-wide autosomal SNP array data to investigate the significance of the genetic legacy of Roma from the populations with which the Romani people could encounter during their long migration towards Europe. The study focuses primarily to the significance of the Caucasus region in Roma ancestry, since it turned out so far to be a rather neglected topic of population genetics. Besides investigating the paternal and maternal lineages, the testing of autosomal data enables the potential to simultaneously analyze multiple genealogies, which can provide additional information about the genetic legacy of Roma, therefore providing also a more complete insight to their history.

## Results

### Population structure, ancestry analysis and F_st_ calculations

We applied population structure and clustering analysis using SMARTPCA and ADMIXTURE to study the relationship of the investigated populations. Throughout the analyses we grouped the populations into regional groups described in the Materials and Methods section.

**Fig 1:**
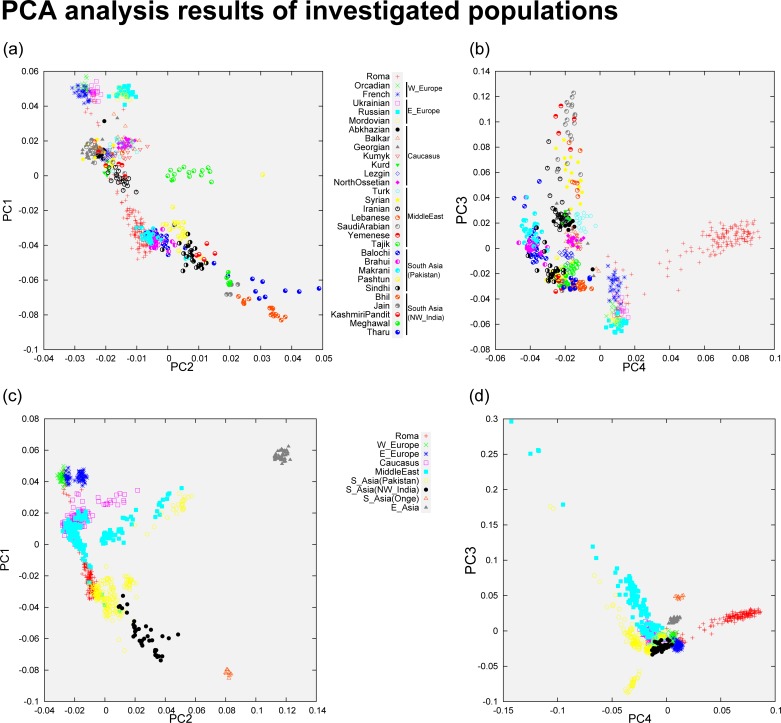
PCA analysis results featuring all populations. Each symbol represents an individual. (a) Shows the population structure of investigated major regional populations on principal components 1 and 2. (b) Shows the population structure of major populations on principal component 2 and 4. (c) All populations included in PCA grouped into regions on principal component 1 and 2 (d) Structure of all populations included in the PCA plotted on principal component 3 and 4. Note that all four graphs are the result of the same PCA. Eigenvalues of PC1 and PC2 were 19.38 and 6.67, eigenvalues of PC3 and PC4 were 6.07 and 5.31.

PCA analysis show that our investigated regional populations form three major groups (Fig 1). West and East European groups formed the somewhat loose European cluster, South Asians formed another group which tends to be much tightly clustered. Populations living in the Caucasus region and in the Middle East are very tightly clustered and located precisely between the European and South Asian clusters. Because of a significant East Asian ancestry, Central Asians separated from their Middle Eastern neighbors. Together with a few South Asian populations, possessing also considerable amount of East Asian ancestry, formed a very loose fourth cluster directed towards the East Asians. Onge are tightly clustered and are separated from the rest of the populations, showing that they do not have recent West Eurasian ancestry components, and shows also East Asian ancestry to some degree. Roma samples are scattered throughout the line defined by three clusters, plotted more frequently near to the South Asian cluster. PCA placed the Roma scattered between Europe and their ancestral home (South Asia), with a more significant relationship with South Asians.

**Fig 2:**
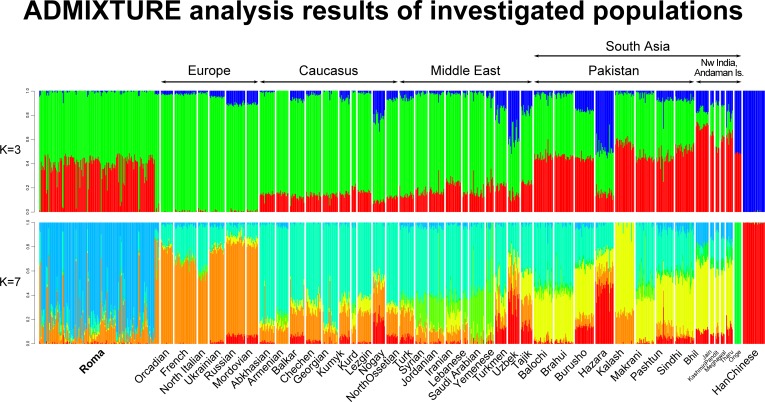
ADMIXTURE analysis results at K = 3 and 7 hypothetical ancestral groups. Each column group represents one population, each column represents one individual. The number of individuals in this analysis was restricted to a maximum size of 30, except of Roma. A figure containing all ADMIXTURE graphs from K = 3 to K = 10 can be found in the supplemental material (S1 Fig).

ADMIXTURE results at K = 3 and 7 hypothetical ancestral groups show similar results (Fig 2). The cross-validation error dropped most significantly at K = 3 already, but reached its minimum at K = 7. The three hypothetical groups, shared between the investigated populations, represent the Western, Central and Eastern part of the Eurasian supercontinent. The transition of genetic composition can be well-observed in both graphs, as the share of hypothetical ancestral groups are gradually changing from Europe to South Asia. The more significant East Asian component shown on the PCA can be also observed on the ADMIXTURE graphs in case of Central Asian and South Asian populations.

**Fig 3:**
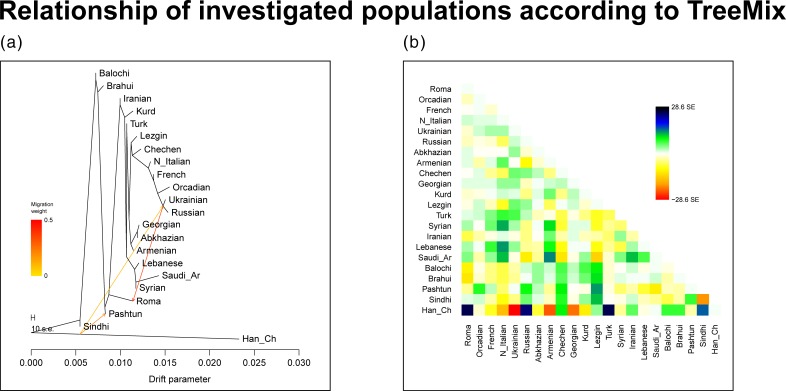
TreeMix analysis results. (a) Maximum likelihood graph and 3 included migration events estimated by TreeMix. Contains population from all investigated regions. (b) The residual fit from the ML graph.

TreeMix placed the Roma between South Asia, Middle East and between the Caucasus region on the maximum likelihood tree, strengthening the results of PCA and ADMIXTURE analysis (Fig 3). One of the 3 included migration events show a strong gene flow from East Europeans into the Roma, and the two other migration events estimates a weaker gene flow from East Asia into South Asia and also into East Europe.

Applying the SMARTPCA software, pairwise average allele frequency differentiation (F_st_) was also calculated between the Roma and investigated regional populations (Table 1). Roma showed the lowest F_st_ values with the populations of the Caucasus region (0.014 on average). F_st_ values regarding Europe and the Middle East was similarly low as in case of the Caucasus region (0.016 on average). The average F_st_ was the highest with South Asians (0.023), but in case of ethnic groups living in the neighboring area of Northwest India e.g. Pashtun and Sindhi, or Northwest Indian populations e.g. Kashmiri Pandit, we obtained low F_st_ values of 0.014, 0.017 and 0.016, respectively.

**Table 1:** Pairwise average allele frequency differentiation (F_st_) values between Roma and the investigated regional populations.

	Europe		Caucasus		Middle East (and C. Asia)		South Asia		East Asia	
	Orcadian	**0.020**	Abkhazian	**0.015**	Turk	**0.011**	Balochi	**0.017**	Han Chinese	**0.093**
	French	**0.015**	Armenian	**0.013**	Syrian	**0.014**	Brahui	**0.018**		
	North Italian	**0.015**	Balkar	**0.013**	Jordanian	**0.015**	Burusho	**0.020**		
	Ukrainian	**0.015**	Chechen	**0.016**	Iranian	**0.013**	Hazara	**0.030**		
	Russian	**0.016**	Georgian	**0.015**	Lebanese	**0.016**	Kalash	**0.040**		
	Mordovian	**0.016**	Kumyk	**0.012**	Saudi Arabian	**0.022**	Makrani	**0.017**		
		** **	Kurd	**0.015**	Yemenese	**0.018**	Pashtun	**0.014**		
		** **	Lezgin	**0.015**	Turkmen	**0.018**	Sindhi	**0.017**		
		** **	Nogay	**0.013**	Uzbek	**0.021**	Bhil	**0.029**		
		** **	North Ossetian	**0.015**	Tajik	**0.013**	Jain	**0.028**		
		** **		** **		** **	Kashmiri Pandit	**0.016**		
		** **		** **		** **	Meghawal	**0.023**		
		** **		** **		** **	Tharu	**0.025**		** **
**Avg.**		**0.016**		**0.014**		**0.016**		**0.023**		**0.093**

### Evidence of admixture and estimated proportions

In order to formally test whether Roma are admixed with the investigated regional populations we constructed two setups for the 4-population test. In the first setup Roma to South Asian populations and investigated regional populations to Han Chinese were related. Onge is an indigenous population of the Andaman and Nicobar Islands in India, and are the only known South Asian population, which does not have recent admixture with populations from the Western area of Eurasia, therefore represents accurately the ancestral South Asian ancestry of Roma [20]. Other applied South Asian populations have some degree of West Eurasian ancestry and can be found in Northwest India and the Pakistani area neighboring with Northwest India, from which area the Romani people originate. [15, 16] These tests showed significant violation in these setups, particularly in case of Onge and some Northwest Indian populations, Meghawal, Tharu and Bhil. Therefore our tests confirmed that Roma and the investigated regional populations are indeed admixed (S1 Table).

To estimate the proportion of the contribution of West Eurasian ancestry in Roma, we applied the F_4_ ratio estimation algorithm from the ADMIXTOOLS Software Package. Our results showed that Roma have a high extent of ancestry from the Western region of Eurasia. Roma show an even higher extent of West Eurasian ancestry in case if we apply groups from the Caucasus region or from the Middle East, than Europeans (CEU) showed in previous studies dealing with the origin of the Romani people, which was 77.5 +/- 1.8% and 81.08 +/- 0.53% [15, 16] (Table 2).

**Table 2:** F4 ratio estimation results. The distribution of South Asian (Indian) ancestry and the ancestry related to investigated regional populations in Romani people.

Group A	Outgroup (O)	Admixed group (X)	Group C	Group A	Outgroup (O)	Group B	Group C	Alpha	Std. Err.	Z-score
WestEuropean	HanChinese	Roma	Onge	WestEuropean	HanChinese	Abkhazian	Onge	0.8882	0.0053	168.40
WestEuropean	HanChinese	Roma	Onge	WestEuropean	HanChinese	Armenian	Onge	0.8575	0.0051	167.91
WestEuropean	HanChinese	Roma	Onge	WestEuropean	HanChinese	Balkar	Onge	0.9637	0.0067	144.57
WestEuropean	HanChinese	Roma	Onge	WestEuropean	HanChinese	Chechen	Onge	0.9042	0.0058	154.91
WestEuropean	HanChinese	Roma	Onge	WestEuropean	HanChinese	Georgian	Onge	0.8590	0.0051	167.91
WestEuropean	HanChinese	Roma	Onge	WestEuropean	HanChinese	Kumyk	Onge	0.9717	0.0070	139.41
WestEuropean	HanChinese	Roma	Onge	WestEuropean	HanChinese	Kurd	Onge	0.8993	0.0078	115.44
WestEuropean	HanChinese	Roma	Onge	WestEuropean	HanChinese	Lezgin	Onge	0.8964	0.0057	157.86
WestEuropean	HanChinese	Roma	Onge	WestEuropean	HanChinese	NorthOssetian	Onge	0.9700	0.0067	144.99
WestEuropean	HanChinese	Roma	Onge	WestEuropean	HanChinese	Turk	Onge	0.9473	0.0060	156.92
WestEuropean	HanChinese	Roma	Onge	WestEuropean	HanChinese	Syrian	Onge	0.8931	0.0059	152.25
WestEuropean	HanChinese	Roma	Onge	WestEuropean	HanChinese	Jordanian	Onge	0.9123	0.0057	159.36
WestEuropean	HanChinese	Roma	Onge	WestEuropean	HanChinese	Iranian	Onge	0.9558	0.0061	156.92
WestEuropean	HanChinese	Roma	Onge	WestEuropean	HanChinese	Lebanese	Onge	0.8960	0.0068	131.03
WestEuropean	HanChinese	Roma	Onge	WestEuropean	HanChinese	SaudiArabian	Onge	0.8997	0.0059	151.25

**Fig 4:**
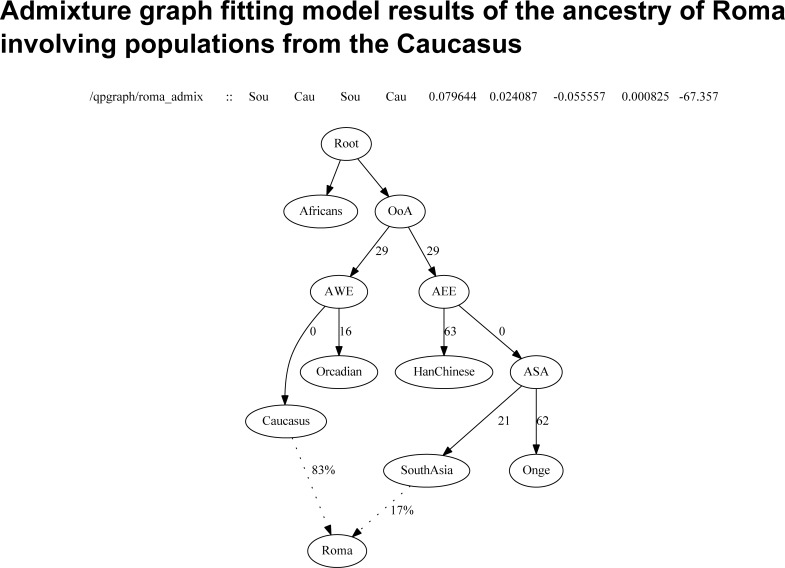
Model setup of the proposed admixture between Roma ancestors and Caucasus populations tested with Admixture graph fitting. Branch lengths are shown in units of F_st_*1000. Worst F-statistics result is shown above the graph. OoA—Out of Africa, AWE—Ancestral West Eurasians, AEE—Ancestral East Eurasians, ASA—Ancestral South Asians.

In order to test further the supposed admixture between Roma and populations from the Caucasus, we investigated a graph model setup with an admixture event using qpGraph from the ADMIXTOOLS software package. Results show that our model fits the data (Fig 4). Estimates of the admixture graph fitting algorithm are similar but slightly lower (83%) regarding the admixture proportion of West Eurasians, which here represented by populations from the Caucasus region.

### IBD analyses

To assess the impact of investigated regions on the Roma ancestry and to study the significance of the Caucasus on the ancestry of Roma, we calculated the average pairwise IBD sharing in case of each regions (South Asia, Middle East and Central Asia and Caucasus).

**Fig 5:**
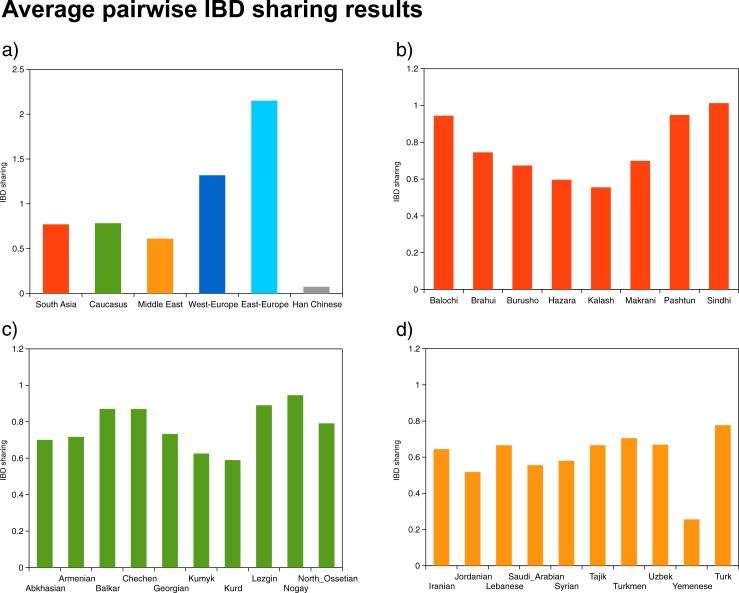
Average pairwise IBD sharing between Roma and the investigated regional populations. (a) IBD share between Roma and investigated regions. (b) IBD share between Roma and South Asian populations. (c) IBD share of Roma with the Caucasus. (d) IBD share of Roma with Middle East and Central Asian populations.

According to our results, the average pairwise IBD sharings in case of Europeans and South Asians correspond to the results reported in previous papers dealing with the source of West Eurasian and South Asian ancestries of Roma [15]. Average IBD sharing of Roma with West Europeans was 1.32, while sharing with East Europeans was 2.15. Average share with South Asians was 0.77 on average, and in case of Balochi, Pashtun and Sindhi it approached 1.00. The share with Middle Eastern and Central Asian populations were less significant with an 0.61 share on average, and approached or exceeded the value 0.70 only in case of some Central Asian populations (Tajik, Turkmen, Uzbek) and in case of Turks. However the IBD share between Roma and the Caucasus was very similar to IBD share of Roma with South Asians with an average value of 0.78. The highest IBD sharings approached 0.9 in case of Balkar, Lezgin and Nogay samples (Fig 5).

**Fig 6:**
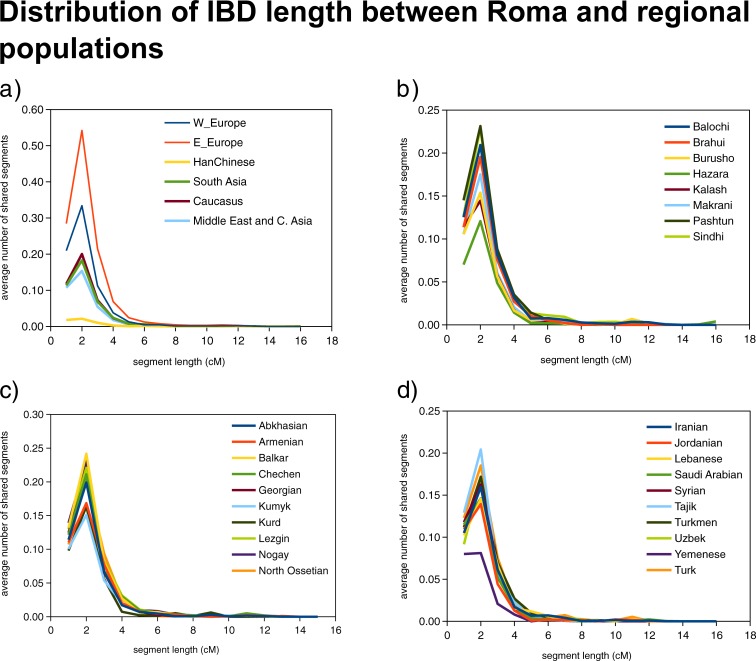
Average IBD length distribution between Roma and all investigated regions. (a) Average IBD length distribution between Roma and all regions. (b) Average IBD length distribution of Roma with South Asian groups. (c) Average IBD length distribution of Roma with the Caucasus. (d) Average IBD length distribution of Roma with populations from the Middle East and Central Asia.

Investigating the IBD length distribution differences between these populations, we obtained results that show, the number of long IBD segments shared with Roma was the highest in case of Europeans as one can expect. We found the lowest number of long IBD segments in the case of Middle Eastern and Central Asian populations. The results in case of South Asians and Caucasus region populations was fairly similar (Fig 6).

### Investigating admixture dates with populations on the migration route

In order to further support the evidence of admixture between Roma and regional populations, and to attempt to determine the date of gene flow between these populations, we applied the algorithm of ALDER. In most of the cases, ALDER could find sufficient correlation between the LD of the references and the test population and could provide significant weighted LD curves to confirm admixture and provide an admixture date estimation. These estimations are summarized in Table 3 and the corresponding diagrams showing the weighted LD plots against genetic distance can be found in the supplemental material (S2 Fig). ALDER calculations show that the date of gene flow in case of the Caucasus region is almost identical to the estimated date of admixture with Middle Eastern and Central Asian populations, however the admixture with populations from the Caucasus is more recent. According to ALDER, the oldest gene-flow event among the investigated regions was the admixture with certain South Asians (Balochi, Brahui, Makrani).

**Table 3:** Admixture date estimation results computed using ALDER.

Region	Population	Est. date of admixture (generations ago)	Std. Dev.	Estimated date of admixture (years ago)		Average estimated date of admixture (years ago)	
				lower limit	upper limit	lower limit	upper limit
**Caucasus**	Abkhasians	32.26	5.61	772.85	1098.23	756.81	1076.45
	Armnenians	34.71	5.24	854.63	1158.55		
	Balkars	30.78	5.53	732.25	1052.99		
	Chechens	32.33	6.61	745.88	1129.26		
	Georgians	31.04	4.58	767.34	1032.98		
	Kumyks	32.85	5.55	791.70	1113.60		
	Kurds	29.76	4.56	730.80	995.28		
	Lezgins	32.40	5.10	791.70	1087.50		
	Nogays	29.23	6.59	656.56	1038.78		
	N. Ossetians	30.72	5.74	724.42	1057.34		
**Middle East and C. Asia**	Turks	30.40	4.86	740.66	1022.54	820.62	1094.96
	Iranians	34.17	5.83	821.86	1160.00		
	Jordanians	34.65	4.36	878.41	1131.29		
	Lebanese	37.11	4.22	953.81	1198.57		
	Saudi Arab.	29.49	4.65	720.36	990.06		
	Syrians	28.93	3.97	723.84	954.10		
	Yemenese	36.44	5.22	905.38	1208.14		
**South Asia**	Balochi	40.78	5.34	1027.76	1337.48	997.70	1299.88
	Brahui	38.75	4.91	981.36	1266.14		
	Makrani	39.31	5.38	983.97	1296.01		

## Discussion

PCA and ancestry analysis with ADMIXTURE placed the Roma on an Eurasian perspective and showed that the investigated regional populations form three major groups, the rather loose groups of Europeans and South Asians and a tightly clustered group constituted by Middle Eastern and Caucasus related populations. Central Asians (but also some of the South Asians) are somewhat outliers due to their high East Asian ancestry proportion. Plotting Roma samples to these populations, Roma are scattered between the three major groups defined by our population structure and ancestry analyses. Most Roma samples can be found between the Middle East, the Caucasus and South Asia, more closely to South Asian samples. TreeMix showed similar results placing Roma between the Middle Eastern populations, the Caucasus region and between South Asians.

F_st_ calculations showed results almost as expected. Roma have the highest F_st_ with South Asian populations and the closer the investigated populations to Europe are, the lower the value of F_st_ gets. However, we found a minimum F_st_ at samples from the Caucasus region and Turkey, which slightly disrupts this trend, suggesting that this region could play an important role in the ancestry of Romani people. The low F_st_ with Turks can be the result that Roma were living in the neighborhood of the Anatolian Peninsula, more precisely on the Balkans for a relatively long period of time, approximately from the 12^th^ to the dawn of the 15th centuries. Their entering into Europe was the result of fleeing from the increasing pressure of conquest campaigns launched by the Ottoman Empire from Anatolia. Also, our Roma data are originating from once Ottoman occupied areas of Europe, mostly from East-Central Europe. Because some of the populations from the Caucasus region show similarly low F_st_, it can indicate the more important role of the area of the Caspian and Black Seas in Roma ancestry.

Although, we investigated the relationship of Roma with regional populations, we had also to formally confirm that they are indeed admixed with each other. Therefore we applied two types of formal tests of admixture. D-statistics test is the one that can prove only the fact that the investigated populations are admixed to some extent, and F_4_ ratio estimation is also able to estimate the admixture proportions of two populations shaping the ancestry of the admixed population, here the Romani people. D-statistics confirmed that Caucasus, Middle East, Central Asia are admixed with the ancestors of Roma, therefore participated in the shaping of Roma ancestry. F_4_ Ratio estimation also confirmed that these populations have a high extent of ancestry proportion to Roma, but they gave similar results, because F_4_ ratio estimation cannot distinguish between distinct ancestries from the Western Eurasian region. Regional populations gave slightly higher results than Europeans, supposedly because these populations are closer to South Asia, and admixture between these populations occurred multiple times subsequently also before the Roma exodus from India. Admixture graph fitting showed that our model for the ancestry of Roma fits to the data therefore confirmed also the supposed admixture between Caucasus populations.

We conducted IBD estimations to assess the contribution of the Caucasus, Middle East and Central Asia and South Asia to the ancestry of Roma. The average pairwise IBD shares pointed out that Caucasus is an important source of Roma ancestry besides South Asia and Europe, therefore confirming that Roma ancestry derived from the Caucasus is significant. Populations of the Caucasus region show slightly higher IBD share than Middle Eastern and Central Asian populations, similar to the share of South Asian populations. This reflects that not only the Middle East but also the Caucasus region could also play important role in Roma ancestry. Our IBD length distribution analyses showed that the Caucasus region has a greater number of shared long IBD segments than Middle East and Central Asia, which suggests that Caucasus populations have admixed with Roma people more recently and have a higher proportion in Roma ancestry.

In order to infer the actual admixture dates of Roma ancestors with the populations of the Caucasus region, Middle East, Central Asia and South Asia, we conducted analyses using ALDER. ALDER shows results as expected. Roma admixture with South Asians is the oldest admixture event occurred during the migration of Roma. Interestingly, ALDER placed the admixture event of the ancestry of Roma with Caucasus and the Middle East to a similar date, but admixture with the Caucasus seems to be slightly more recent. These admixture dates fit well into results of different scientific fields and also into the results of genetic studies estimating the exodus of Roma from the Indian subcontinent and their arrival into Europe. Besides a tool for admixture date estimation, ALDER also serves as a robust and highly unbiased method to test whether two investigated population are admixed with each other. Therefore we obtained another confirmation for the supposed admixture events with a method based on admixture-LD instead of the method based on allele frequency data.

Applying genome-wide autosomal marker data, we were able to assess the contribution of the Caucasus to the ancestry of Roma, which clearly seems to be significant compared to the two main sources of their ancestry, Europe and the population of their area of origin, South Asia. These analyses show that the Caucasus region can be the most important source of Roma ancestry taking into consideration their migration route, which also included Central Asia and the Middle East. Our results suggest that the area of the Caspian and Black Seas is a significant source for the genetic legacy of Romani people, which suggests that the region, therefore also the Caucasus plays and important role in their ancestry and migration history.

## Materials and methods

### Datasets

Our Roma samples comprised of two datasets. The first Roma dataset (n = 27; 726,016 SNPs) was collected and genotyped in international collaboration and was described previously in two studies [15, 16]. The second Roma dataset (n = 152, 868,174 SNPs) was obtained from an upon request available source and was described also previously in a paper dealing with the origin of Roma people [14]. The first dataset was genotyped on a custom Affymetrix 1 M chip based on the Affymetrix Genome-Wide Human SNP array 6.0 platform, and the second dataset was genotyped on the Affymetrix Genome-Wide Human SNP array 6.0 chip. The datasets contained Roma samples from all across Europe, mainly from East-Central Europe and from the Iberian Peninsula. We conducted preliminary population structure analyses in order to remove Roma samples from the larger and more diverse data introduced in *Mendizabal et al*. *2012*, in which there are some samples heavily admixed with non-Roma Europeans (mainly from the Iberian Peninsula), therefore creating an outlier subgroup in our data (S3 Fig). After the sample removal, the merged and Roma data contained 158 Roma samples featuring 599,471 SNPs.

In this study we considered various datasets in order to investigate the significance of the Caucasus region in the history of Roma. We applied samples from the CEPH-Human Genome Diversity Panel (HGDP) (n = 1044 from 57 populations, 660,918 SNPs genotyped on Illumina 650 Y array). We also used two publicly available datasets of the Estonian Biocentre described in two distinct papers [21, 22]. We will refer to these two datasets simply as “Caucasus data” (n = 204 from 13 populations, 555,767 SNPs) and “Jew data” (n = 466 from 39 populations, 555,736 SNPs) from now on. The last dataset we considered was the upon request available “India data” (n = 121 from 23 ethnic groups, 524,053 SNPs, genotyped on Affymetrix 1 M and Illumina 650K arrays) described previously in a paper dealing with the population history of India [20].

We extracted most of the European populations (Orcadian, French, North Italian, Russian) and non-Indian South Asian populations (Balochi, Brahui, Burusho, Hazara, Kalash, Makrani, Pashtun, Sindhi) from the HGDP data. Han Chinese as an East Asian outgroup used in our tests was also extracted from the HGDP dataset. Population samples regarding the Caucasus region (Abkhasian, Armenian, Balkar, Chechen, Georgian, Kumyk, Kurd, Lezgin, Nogay, North Ossetian) and the Middle East and Central Asia (Iranian, Jordanian, Lebanese, Saudi Arabian, Syrian, Tajik, Turk, Turkmen, Uzbek, Yemenese) as well as some additional East European data (Ukrainian and Mordovian) were extracted from the Caucasus and Jew data. From the Indian data we applied Northwest Indian populations such as Bhil, Jain, Kashmiri Pandit, Meghawal Tharu and also the Onge living on the Andaman and Nicobar Islands.

### Population structure and ancestry analysis

In order to study the relationship of the previously described regional populations (South Asia, Caucasus, Middle East and Central Asia) with Roma, we implemented three different methods. We performed PCA using the SMARTPCA software from the EIGENSOFT 6.01 Software Package [23] to investigate the structure of all populations in question. SMARTPCA was also used to compute the pairwise average allele frequency differentiation (F_st_) matrix from the data. Besides the mathematical approach, we applied the statistical model based STRUCTURE-like approach, ADMIXTURE to infer to the ancestries of investigated populations. ADMIXTURE implements a maximum likelihood estimation method, which estimates population ancestry distribution in a perspective of a number of hypothetical ancestral groups [24].

We applied also the TreeMix 1.13, which constructs the maximum likelihood (ML) graph of investigated populations based on genome-wide allele frequency data [25]. The plotted ML graph estimated by TreeMix shows population splits, admixture events, and the algorithm can be also set to infer probable migration processes, which can be visualized on the plotted graph.

For PCA and ADMIXTURE analysis, we created a dataset containing Europeans, South Asian, Caucasus, Middle East and Central Asian populations (n = 908 from 43 populations, 123,048 SNPs). For the TreeMix analysis we created a smaller dataset for better visualization, which also contained populations from all investigated regions (n = 584 from 22 populations, 123,048 SNPs).

Since strong background linkage disequilibrium (LD) can adversely affect these analyses, the marker set of the data was thinned using the pairwise genotypic correlation based pruning method of PLINK 1.07 [26]. The pairwise genotypic correlation variable (r^2^) was set to 0.3. Other parameters of the algorithm were at its default settings, since the size of the window was 50 SNPs with a sliding of 5 SNPs at a time. The thinned datasets contained 81,524 SNPs.

SMARTPCA was also used with its default settings, the σ-threshold, which here defines outliers to be removed from the analysis, was set to 6.0. We made a cross-validation error check with ADMIXTURE at K = 2 to K = 12 hypothetical ancestral groups to find the appropriate value of K for the most accurate ancestry distribution estimation. At the TreeMix analysis, we used Han Chinese as root population and set the SNP block size parameter (-k) to 500. Three migration events were also added to the ML tree according to the resulted standard error of the residual fit diagrams.

### Formal test of admixture

We applied the algorithm 4-population test included in the ADMIXTOOLS 4.1 Software Package [27] for the formal test, whether Roma and regional populations in our focus are truly admixed with each other. The algorithm qpDstat incorporated in the software package applies the 4-population test method, implemented as D-statistics, for formal test of admixture.

In order to reveal admixture between Roma and regional populations, we used the unpruned dataset of SMARTPCA and ADMIXTURE analysis. For testing admixture between Roma and regional populations (South Asia, Caucasus, Middle East and Central Asia), we computed the D-statistics of the unrooted phylogenetic trees ((Roma, South Asians)(Regional Populations, Han Chinese)). The test shows us whether Roma and regional populations on the migration route of Roma are admixed.

We attempted also to measure the proportion of Central Asian, Middle Eastern and Caucasus region derived ancestry in Roma, therefore we applied the F_4_ ratio estimation method of ADMIXTOOLS. We investigated the ratio of f_4_(West Europeans, Han Chinese, Roma, Onge)/f_4_(West Europeans, Han Chinese, Regional populations, Onge). We applied West European populations to represent another branch of West Eurasians besides Caucasus populations and Middle East on the hypothetical ancestry graph and Onge represented the South Asian ancestry of Roma.

The ADMIXTOOLS Software package also contains the Admixture graph fitting algorithm. This algorithm allows one to build and test models of population relationships based on allele frequency correlations. It is similar to the TreeMix algorithm, except it does not offer best fits to the data from possible models, rather it provides a rigorous test method for investigating a particular concept of a supposed graph of population relationships. In this test we applied Caucasus populations, South Asian populations, Orcadians and Han Chinese to test the hypothesis that recent Roma are admixed with Caucasus populations. Our model graph separates the Eurasian populations into two branches. Caucasus populations belong to the Western branch with Orcadians on a separate sub-branch and South Asian populations belong to the Eastern branch with Onge on a separate sub-branch. The reason of the sub-branches is that Orcadian samples show a relatively high F_st_ with Roma according the calculations of SMARTPCA. On the other hand, Onge do not possess recent West Eurasian ancestry, in contrast of other South Asians applied besides them here in the tests. Han Chinese form an outgroup on the Eastern branch. On the model, Caucasus populations contributed to the ancestry of recent Roma, which is shown as an admixture event between Caucasus populations, and the ancestors of Roma, surrogated here by South Asian populations. F-statistics were normalized by heterozygosity in the applied outgroup Han Chinese.

### Identity-by-descent segment analyses

For the assessment of the extent of South Asian, Caucasus region, Middle Eastern and Central Asian ancestry of Roma, we applied the Refined IBD algorithm of Beagle 4.1 [28]. Using the software, we searched for identical by descent (IBD) segments between Roma and populations classified into regions. Here we applied also the dataset that was previously used in the population structure and ancestry analyses, however we submitted the full marker set to the IBD estimation algorithm without the LD-based pruning (n = 908 from 43 populations, 123,048 SNPs). Before the analysis, the data was formatted accordingly. Major alleles were set as A1 allele with PLINK 1.07 and the dataset packed into binary file format was converted to Variant Call Format 4.1 using the PLINK/SEQ 0.10 package [29]. We set the minimum segment length to 3 cM, the IBD trim parameter setting to 10, and applied the recommended setting as the IBD scale parameter. The recommended value of the IBD trim parameter is $\sqrt{n/100}$, if the dataset contains more than 400 individuals, otherwise one should use the value of 2 [28].

We calculated an average pairwise IBD sharing between Roma (population I) and regional populations (population J) from the output of Beagle:

$$Average\;pairwise\;IBD\;sharing=\frac{\sum_{i=1}^{n}\sum_{j=1}^{m}{IBD}_{ij}}{n∙m}$$

where IBD_ij_ is the length of IBD segment shared between individuals i and j, and n, m are the number of individuals in population I and J [30].

Based on the fact that higher number of long IBD segments implies more recent admixture between two populations, we investigated the issue, whether there is a detectable difference between the dates of admixture of Roma with distinct regional populations on Roma migration route. We calculated the distribution of the average number of IBD lengths between pairs of individuals from Roma and regional populations. IBD segments were classified based on their lengths, the number of segments were counted in case of each class and were divided with the number of all possible pairs of individuals.

### ALDER analysis

Besides the IBD length analysis we attempted to infer the chronology and the exact date of the gene flow between Roma and regional populations implementing the ALDER 1.03 algorithm [31]. ALDER is capable to estimate the date of population admixture. Like its predecessor ROLLOFF, it is also based on the decay of LD caused by an admixture event. The algorithm computes correlations between SNPs in an admixed target population weighted according to the allele frequency difference in ancestral populations. Latter serve as reference populations to the algorithm. The results are highly affected by admixture LD, and the algorithm uses allele frequency values in the ancestral populations to amplify the signal of LD caused by an admixture event, which helps in filtering out the background LD. The enhanced algorithm of ALDER provides more sophisticated weighted LD statistics, has the ability to totally avoid biased estimates caused by background LD, and a further major advantage is that it is capable to use the target population itself as reference leading to virtually unbiased statistics.

We estimated the date of admixture between Roma and regional populations using the same dataset that was used also in D-statistics. We ran separate 2-reference tests with ALDER to obtain weighted LD values. In these separate tests, Onge and one population from South Asia, the Caucasus and Middle East were used as reference populations, and the target population was the Roma.

## Supporting information

S1 Fig ADMIXTURE analysis results using 3–10 hypothetical ancestral groups. (EPS)

S2 Fig ALDER analysis diagrams. Weighted LD values plotted against the genetic distance.
(PDF)

S3 Fig PCA analysis of Roma datasets before and after the sample removal. Roma01—Roma data from *Mendizabal et al*. *2012*, Roma02—Roma data from *Moorjani et al*. *2013*.
(EPS)

S1 Table D-statistics results of Roma and regional population. Formal test of admixture applying D-statistics.
(PDF)
