# Supplementary material for: Revealing the impact of the Caucasus region on the genetic legacy of Romani people from genome-wide data
Source: PLoS One. 2018 Sep 10;13(9):e0202890. doi: 10.1371/journal.pone.0202890 (PMC6130880; doi:10.1371/journal.pone.0202890)

Plotting weighted LD against genetic distance computed by ALDER

Caucasus

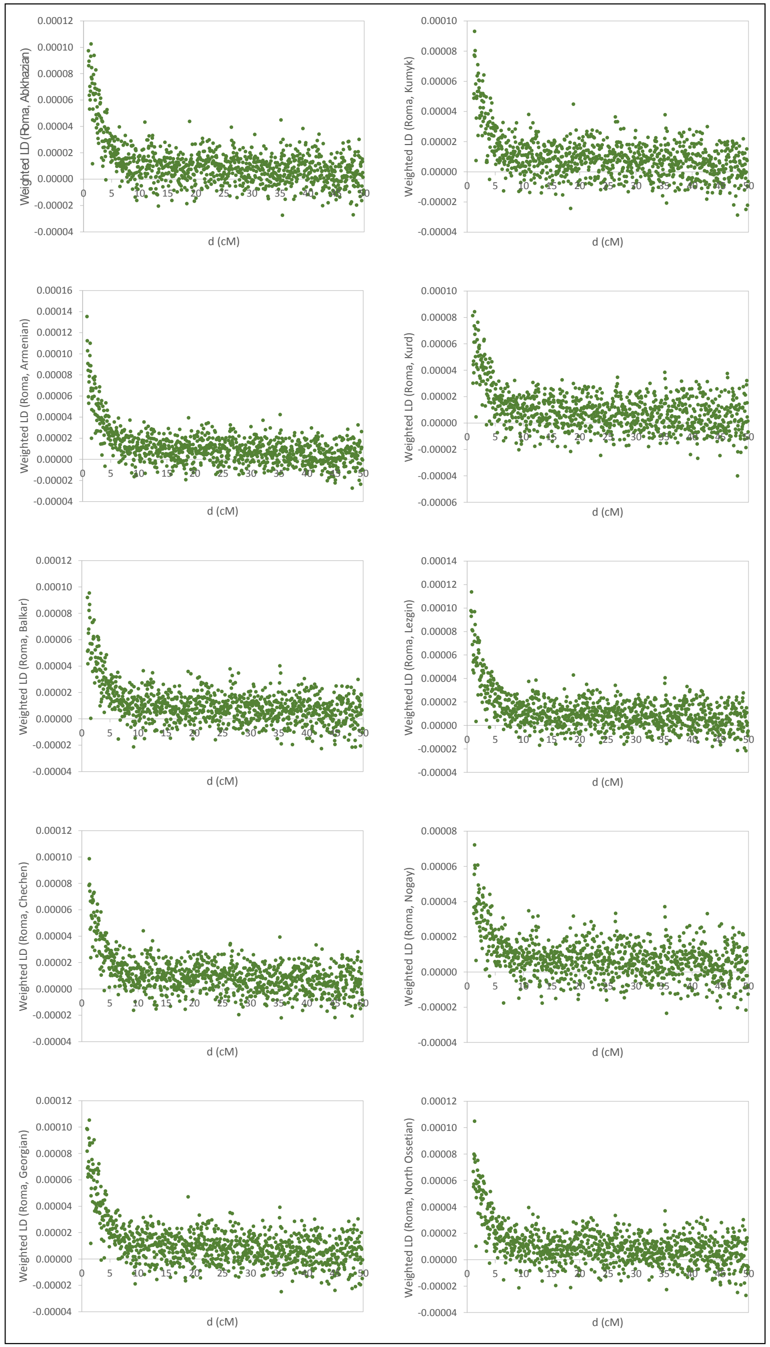

Middle East

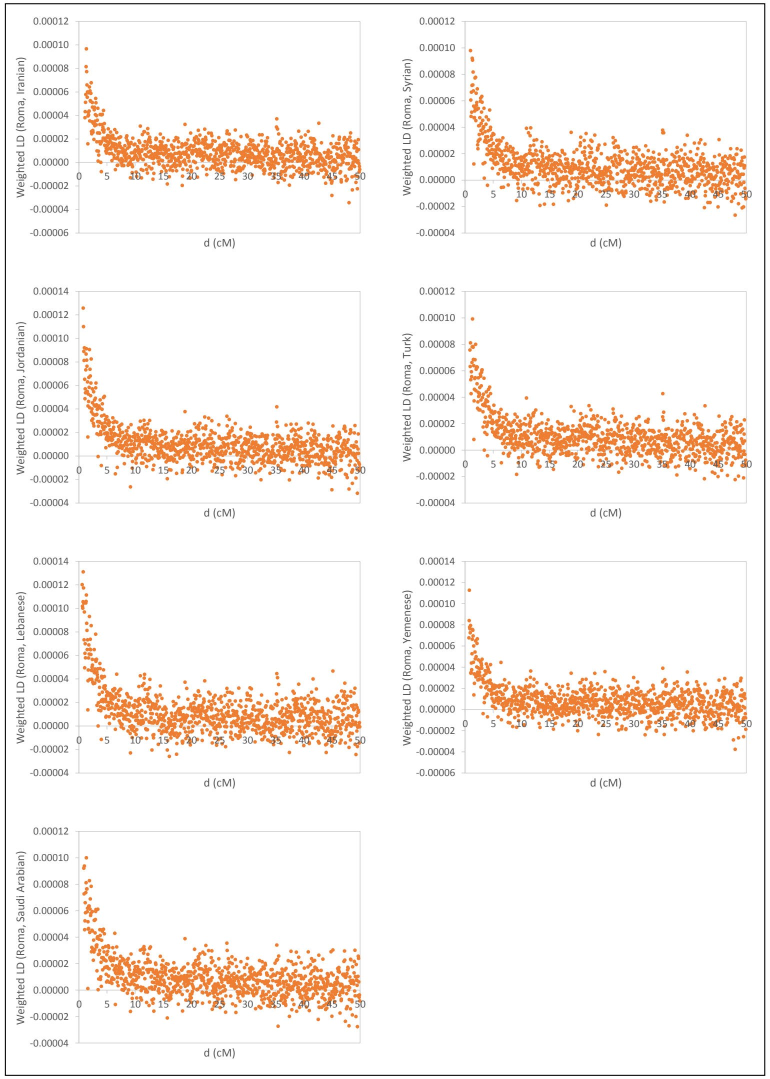

South Asia

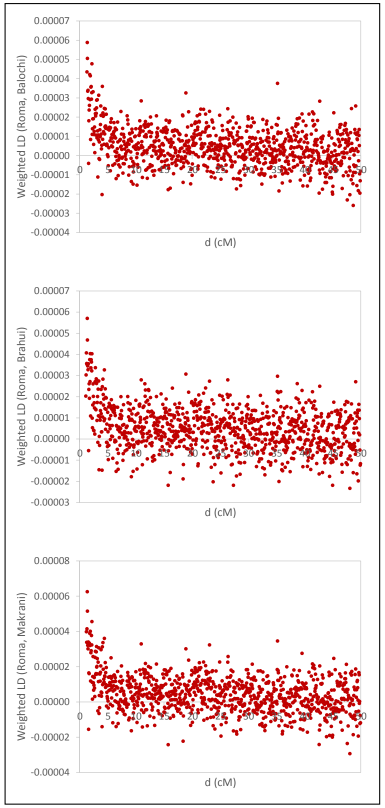

Supplement: S2 Fig — Weighted LD values plotted against the genetic distance. (PDF) [file pone.0202890.s002.pdf]
