# Supplementary material for: Revealing the impact of the Caucasus region on the genetic legacy of Romani people from genome-wide data
Source: PLoS One. 2018 Sep 10;13(9):e0202890. doi: 10.1371/journal.pone.0202890 (PMC6130880; doi:10.1371/journal.pone.0202890)
Supplement: S1 Table — Formal test of admixture applying D-statistics. (PDF) [file pone.0202890.s004.pdf]

Region	Populations					Results	
	W	X	:	Y	Z	D-statistics	Z-score
**Caucasus**	Roma	Onge	:	Abkhazian	HanChinese	0.132	56.004
	Roma	Onge	:	Armenian	HanChinese	0.133	54.829
	Roma	Onge	:	Balkar	HanChinese	0.125	55.259
	Roma	Onge	:	Chechen	HanChinese	0.129	56.423
	Roma	Onge	:	Georgian	HanChinese	0.134	56.557
	Roma	Onge	:	Kumyk	HanChinese	0.125	54.232
	Roma	Onge	:	Kurd	HanChinese	0.128	52.427
	Roma	Onge	:	Lezgin	HanChinese	0.130	57.395
	Roma	Onge	:	Nogay	HanChinese	0.108	53.502
	Roma	Onge	:	NorthOssetian	HanChinese	0.126	54.900
**Middle East**	Roma	Onge	:	Turk	HanChinese	0.127	57.074
	Roma	Onge	:	Jordanian	HanChinese	0.126	54.276
	Roma	Onge	:	Iranian	HanChinese	0.122	53.225
	Roma	Onge	:	Lebanese	HanChinese	0.129	50.817
	Roma	Onge	:	SaudiArabian	HanChinese	0.126	51.918
	Roma	Onge	:	Yemenese	HanChinese	0.111	45.800
**Caucasus**	Roma	Pashtun	:	Abkhazian	HanChinese	0.019	20.480
	Roma	Pashtun	:	Armenian	HanChinese	0.021	22.548
	Roma	Pashtun	:	Balkar	HanChinese	0.019	20.746
	Roma	Pashtun	:	Chechen	HanChinese	0.018	19.757
	Roma	Pashtun	:	Georgian	HanChinese	0.020	20.997
	Roma	Pashtun	:	Kumyk	HanChinese	0.018	19.655
	Roma	Pashtun	:	Kurd	HanChinese	0.019	19.195
	Roma	Pashtun	:	Lezgin	HanChinese	0.017	18.065
	Roma	Pashtun	:	Nogay	HanChinese	0.016	20.752
	Roma	Pashtun	:	NorthOssetian	HanChinese	0.018	19.612
**Middle East**	Roma	Pashtun	:	Turk	HanChinese	0.020	22.611
	Roma	Pashtun	:	Jordanian	HanChinese	0.022	24.817
	Roma	Pashtun	:	Iranian	HanChinese	0.016	18.329
	Roma	Pashtun	:	Lebanese	HanChinese	0.023	23.493
	Roma	Pashtun	:	SaudiArabian	HanChinese	0.024	25.381
	Roma	Pashtun	:	Yemenese	HanChinese	0.021	21.466
**Caucasus**	Roma	Sindhi	:	Abkhazian	HanChinese	0.022	22.495
	Roma	Sindhi	:	Armenian	HanChinese	0.024	23.399
	Roma	Sindhi	:	Balkar	HanChinese	0.022	22.896
	Roma	Sindhi	:	Chechen	HanChinese	0.022	22.045
	Roma	Sindhi	:	Georgian	HanChinese	0.023	22.749
	Roma	Sindhi	:	Kumyk	HanChinese	0.021	21.753
	Roma	Sindhi	:	Kurd	HanChinese	0.020	20.175
	Roma	Sindhi	:	Lezgin	HanChinese	0.020	20.589
	Roma	Sindhi	:	Nogay	HanChinese	0.020	23.014
	Roma	Sindhi	:	NorthOssetian	HanChinese	0.021	21.312
**Middle East**	Roma	Sindhi	:	Turk	HanChinese	0.023	23.789
	Roma	Sindhi	:	Jordanian	HanChinese	0.023	24.207
	Roma	Sindhi	:	Iranian	HanChinese	0.017	18.586
	Roma	Sindhi	:	Lebanese	HanChinese	0.024	23.582
	Roma	Sindhi	:	SaudiArabian	HanChinese	0.024	24.766
	Roma	Sindhi	:	Yemenese	HanChinese	0.018	18.165
**Caucasus**	Roma	KashmiriPandit	:	Abkhazian	HanChinese	0.029	20.385
	Roma	KashmiriPandit	:	Armenian	HanChinese	0.032	21.916
	Roma	KashmiriPandit	:	Balkar	HanChinese	0.028	20.517
	Roma	KashmiriPandit	:	Chechen	HanChinese	0.028	20.259
	Roma	KashmiriPandit	:	Georgian	HanChinese	0.031	20.920
	Roma	KashmiriPandit	:	Kumyk	HanChinese	0.028	20.521
	Roma	KashmiriPandit	:	Kurd	HanChinese	0.028	18.340
	Roma	KashmiriPandit	:	Lezgin	HanChinese	0.027	18.755
	Roma	KashmiriPandit	:	Nogay	HanChinese	0.025	20.706
	Roma	KashmiriPandit	:	NorthOssetian	HanChinese	0.028	19.957
**Middle East**	Roma	KashmiriPandit	:	Turk	HanChinese	0.030	22.277
	Roma	KashmiriPandit	:	Jordanian	HanChinese	0.033	23.722
	Roma	KashmiriPandit	:	Iranian	HanChinese	0.025	18.904
	Roma	KashmiriPandit	:	Lebanese	HanChinese	0.033	22.901
	Roma	KashmiriPandit	:	SaudiArabian	HanChinese	0.035	24.179
	Roma	KashmiriPandit	:	Yemenese	HanChinese	0.031	20.674
**Caucasus**	Roma	Meghawal	:	Abkhazian	HanChinese	0.044	27.832
	Roma	Meghawal	:	Armenian	HanChinese	0.046	29.230
	Roma	Meghawal	:	Balkar	HanChinese	0.043	28.742
	Roma	Meghawal	:	Chechen	HanChinese	0.043	28.356
	Roma	Meghawal	:	Georgian	HanChinese	0.045	28.235
	Roma	Meghawal	:	Kumyk	HanChinese	0.041	27.235
	Roma	Meghawal	:	Kurd	HanChinese	0.042	26.263
	Roma	Meghawal	:	Lezgin	HanChinese	0.042	27.041
	Roma	Meghawal	:	Nogay	HanChinese	0.036	27.009
	Roma	Meghawal	:	NorthOssetian	HanChinese	0.042	27.485
**Middle East**	Roma	Meghawal	:	Turk	HanChinese	0.044	29.192
	Roma	Meghawal	:	Jordanian	HanChinese	0.046	29.946
	Roma	Meghawal	:	Iranian	HanChinese	0.039	25.674
	Roma	Meghawal	:	Lebanese	HanChinese	0.047	28.284
	Roma	Meghawal	:	SaudiArabian	HanChinese	0.046	28.822
	Roma	Meghawal	:	Yemenese	HanChinese	0.040	25.694
**Caucasus**	Roma	Bhil	:	Abkhazian	HanChinese	0.064	47.535
	Roma	Bhil	:	Armenian	HanChinese	0.066	48.039
	Roma	Bhil	:	Balkar	HanChinese	0.062	48.343
	Roma	Bhil	:	Chechen	HanChinese	0.063	48.707
	Roma	Bhil	:	Georgian	HanChinese	0.066	48.257
	Roma	Bhil	:	Kumyk	HanChinese	0.060	46.959
	Roma	Bhil	:	Kurd	HanChinese	0.061	45.421
	Roma	Bhil	:	Lezgin	HanChinese	0.063	48.814
	Roma	Bhil	:	Nogay	HanChinese	0.053	46.842
	Roma	Bhil	:	NorthOssetian	HanChinese	0.061	46.453
**Middle East**	Roma	Bhil	:	Turk	HanChinese	0.063	48.706
	Roma	Bhil	:	Jordanian	HanChinese	0.063	49.105
	Roma	Bhil	:	Iranian	HanChinese	0.057	44.873
	Roma	Bhil	:	Lebanese	HanChinese	0.066	46.352
	Roma	Bhil	:	SaudiArabian	HanChinese	0.065	48.119
	Roma	Bhil	:	Yemenese	HanChinese	0.056	41.107
**Caucasus**	Roma	Tharu	:	Abkhazian	HanChinese	0.066	46.271
	Roma	Tharu	:	Armenian	HanChinese	0.068	45.933
	Roma	Tharu	:	Balkar	HanChinese	0.062	45.585
	Roma	Tharu	:	Chechen	HanChinese	0.064	45.888
	Roma	Tharu	:	Georgian	HanChinese	0.067	45.275
	Roma	Tharu	:	Kumyk	HanChinese	0.061	45.928
	Roma	Tharu	:	Kurd	HanChinese	0.063	42.806
	Roma	Tharu	:	Lezgin	HanChinese	0.063	44.343
	Roma	Tharu	:	Nogay	HanChinese	0.054	43.615
	Roma	Tharu	:	NorthOssetian	HanChinese	0.063	44.398
**Middle East**	Roma	Tharu	:	Turk	HanChinese	0.064	45.380
	Roma	Tharu	:	Jordanian	HanChinese	0.065	46.962
	Roma	Tharu	:	Iranian	HanChinese	0.059	43.079
	Roma	Tharu	:	Lebanese	HanChinese	0.066	42.456
	Roma	Tharu	:	SaudiArabian	HanChinese	0.066	45.908
	Roma	Tharu	:	Yemenese	HanChinese	0.058	40.189
